# Comparison of Imaging Modalities in Differentiating Cerebral Neoplastic Lesions and Post-radiation Necrosis

**DOI:** 10.7759/cureus.78653

**Published:** 2025-02-06

**Authors:** Sehrish Arif, Rajesh C Varma, Sneha Thaiparambil, Akanksha Ahuja, Arun Nair

**Affiliations:** 1 Surgery, New York City (NYC) Health Hospitals, New York City, USA; 2 Pediatrics, Saint Peter’s University Hospital, New Brunswick, USA; 3 Maternal Fetal Medicine, Saint Peter’s University Hospital, New Brunswick, USA; 4 Medicine, St. George's University, University Centre, GRD

**Keywords:** brain tumors, cns tumors, imaging modalities, neuro-oncology, post-radiation effect, radiation-induced brain necrosis

## Abstract

Neuroimaging of cerebral neoplastic lesions and post-radiation necrosis (PRN) presents significant challenges due to their overlapping features, making differentiation difficult. The use of various imaging modalities in association with radiation therapy introduces potential risks and prognostic variations that can affect lesion physiology. Patients who undergo radiation treatment inevitably experience changes influenced by factors such as radiation dose, brain volume, and tumor fraction size. Additionally, vascular injury and the inflammatory response associated with radiation contribute to alterations observed in neuroimaging. This literature review aims to provide a comparative overview of imaging studies to highlight the optimal modality to distinguish between PRN and tumor recurrence. The imaging modalities assessed included dynamic contrast-enhanced (DCE) and dynamic susceptibility contrast (DSC) MR-perfusion, MR-spectroscopy, intravoxel incoherent motion (IVIM) perfusion, and nuclear medicine studies, including 18F-fluoro-ethyl-1-tyrosine positron emission tomography (18F-FET PET) and 11C-methionine PET (11C MET-PET). The improvement of diagnostic accuracy in multimodal imaging must be further investigated to improve clinical patient management and outcomes of tumor reoccurrence.

## Introduction and background

Existing literature has often been debated on the ideal diagnostic modality to differentiate between post-radiation necrosis (PRN) and the recurrence of a cerebral tumor. Much focus has been on magnetic resonance imaging (MRI) with different contrast mediums. Diagnostic methods within the field of neuroradiology have been a growing area of interest, with the constant attempt to discover new methods with increased sensitivity and specificity. Therefore, understandably, multiple studies have looked at various modalities and their efficacy.

Although it is known that the most common central nervous system (CNS) malignant lesions are metastatic cancers and that the most common primary malignant lesions in adults are gliomas [1] and medulloblastoma in the pediatric population [2], it is equally important to investigate the risk factors and pathophysiology of the development of radiation necrosis (RN). Features of the radiotherapy given, especially toward the brain, can determine how likely a patient can develop RN, including the dosage, the volume of brain irradiated, previous history of radiation therapy, and even the use of concomitant systemic therapy [3]. The type of radiation used may also play a role in the development of RN, for example, proton beam therapy has been shown to possibly reduce the incidence of RN compared to photon radiation therapy [4].

In understanding how to differentiate between RN and the recurrence of a cerebral neoplastic lesion, it is imperative to note the differences in the pathophysiology of both conditions. RN is characterized by a multi-step process in development, and the symptoms that ensue can be dependent on time. The fundamental structures involved in the pathophysiology of this condition include vascular structures, progenitor cells (including oligodendrocytes which can lead to demyelination), endothelial cells, and DNA [5]. It is interesting to note that whilst the mechanism is not well understood, proposed theories have suggested that disruption may also play a key role in the development of symptoms, which include signs of cerebral edema, seizures, and somnolence [5].

Due to the chronic inflammation resulting from irradiation, the presence of increased levels of tumor necrosis factor-alpha (TNF-α) results in increased permeability of the blood-brain barrier and endothelial cell apoptosis. It has also been noted that increased expression of vascular endothelial growth factor (VEGF) results in increased permeability of small vessels resulting in the development of cerebral edema [6]. This is one of the distinguishing factors between PRN and an early delayed reaction [6]. Although there are many precipitating theories on how RN occurs, a biopsy is still required for confirmatory diagnosis as it is nearly impossible to differentiate between a recurrent neoplastic lesion and an RN on clinical or radiological examination [7]. The incidence of radiation necrosis still varies greatly as studies have reported a range between 7% (when using pathological confirmation) and 24% (when using imaging) [8].

Due to invasive risks of obtaining a biopsy, radiological methods of distinguishing RN and recurrence of cerebral neoplastic lesion have been explored sparsely through literature. This literature review aims to investigate, from existing literature, the optimal radiographic method to distinguish both conditions and thereby providing a different perspective that may improve patient care.

## Review

Materials and methods

A literature review format was used to evaluate pre-existing research articles on cerebral neoplastic lesions versus PRN to understand the pathophysiology, the rate of recurrences, the various sensitivity and specificity imaging outcomes, and strategies for differentiating the cause of the lesion. Articles reviewed in this study were published after 2001. The data were collected and processed from Cochrane Library, Google Scholar, and Pubmed databases. Keywords used in identifying admissible articles include “cerebral neoplastic lesions”, “post-radiation necrosis", “imaging modalities”, “neuro-oncologic imaging”, “tumor recurrence “, and “radiation therapy.” Articles pertaining to the research question that involved diagnosing either the recurrence of the tumor or diagnosing PRN with the comparative analysis done were included in this study. All studies were compiled on a shared document in which the studies listed were reviewed by the investigators in the study and agreed upon to provide a perspective to the research question. The several investigators involved in the review had confirmed the cited data.

Post-radiation Necrosis

PRN is a potential complication following radiation therapy that has a wide and varied incidence between 5% and 25% [3,9]. It is a known complication following radiotherapy for various intracranial and skull base tumors, including nasopharyngeal carcinoma (NPC), gliomas, brain metastases, and intracranial arteriovenous malformations [6]. RN is noted to be initiated with vascular damage occurring within the first 24 hours post-radiation, followed by injury to the brain parenchyma [10]. Three main hypotheses explain the development of RN including vascular injury, damage to glial cells and white matter, inflammatory responses, and abnormal cytokine expression [10,11]. Ionizing radiation generates reactive oxygen species in tumor cells, causing single- and double-stranded DNA breaks. This activates DNA repair pathways, leading to cell cycle arrest and apoptosis of irreparably damaged DNA. Radiation also affects the cytoplasmic membrane, destroying endothelial cells and inducing ceramide-triggered apoptosis. These events cause swelling, necrosis, further reactive oxygen species production, and inflammatory responses involving cytokines and chemokines. Consequently, fibrin-platelet thrombus formation and fibrinoid necrosis disrupt the blood-brain barrier and lead to brain edema [5].

The criteria defining RN differ among studies, including the requirement for histological confirmation. Factors contributing to this variability include advancements in diagnostic imaging, enhanced awareness and reporting in oncology, and differing durations of patient follow-up [7,12]. For instance, a study reported a 7% incidence of RN, requiring pathological confirmation or temporal resolution [13]. Conversely, a study observed a higher incidence of 24% (14% symptomatic, 10% asymptomatic), primarily based on imaging criteria such as increased contrast enhancement and stability over months, alongside reduced perfusion on dynamic MRI sequences [7]. 

The occurrence and progression of RN depend on cumulative radiation dose, fraction size, and brain volume [5]. Higher total radiation doses, larger fraction sizes, and increased brain volumes correlated with an elevated incidence of RN as well as an earlier onset. Adjuvant chemotherapy following radiotherapy can increase the incidence of RN fivefold. For malignant gliomas, the incidence of RN in patients surviving more than a year post-irradiation is typically 10%-15%. In NPC patients receiving conventional radiotherapy doses below 6,000 cGy), temporal lobe necrosis incidence ranges widely from 1.6% to 22% over nine months to 16 years [12]. Specifically, a 5% incidence of temporal lobe necrosis within 10 years has been noted after conventional segmental radiotherapy for NPC. Notably, according to the University Cancer Center, temporal lobe injuries can affect up to 34.9% of NPC patients undergoing routine radiotherapy [5]. 

Cerebral Neoplastic Lesions

The incidence of brain and CNS tumors has been increasing in recent years with around 308,000 new cases of brain tumors worldwide until 2020 [14]. With the rising number of cases, it is imperative to distinguish true recurrence versus RN however, there is a phenomenon that must be considered which is pseudoprogression. Pseudoprogression can be defined as an interruption in the synthesis of myelin secondary to injury to oligodendrocytes from radiation [6]. The time period in which this may appear can vary from weeks to months and can be seen on an MRI that may also mimic tumor progression [9].

The diagnosis and differentiation of RN from tumor recurrence in patients typically rely on advanced imaging techniques due to the overlapping features observed in conventional MRI. Radiation necrosis can be characterized by a transient or progressive increase in enhancing lesion size due to inflammatory changes and mimics tumor progression on imaging [15]. While surgery is indicated in some cases of radiation necrosis, other cases in which it is mistaken for progression will result in unnecessary procedures or further radiation, which carry their own inherent risks, or suboptimal systemic therapies tailored to true progression. Multiple advanced imaging techniques including dynamic contrast-enhanced (DCE) and dynamic susceptibility contrast magnetic resonance perfusion (DSC-MR), magnetic resonance spectroscopy (MR-S), intravoxel incoherent motion (IVIM) perfusion, and nuclear medicine studies including 18F-fluoro-ethyl-1-tyrosine positron emission tomography (18F-FET PET) and 11C-Methionine positron emission tomography (11C MET-PET) have been applied to differentiate radiation necrosis from tumor progression [15].

Comparison of Imaging Modalities in Differentiating Tumor Recurrence and PRN

The diagnosis and differentiation of RN from tumor recurrence in patients typically rely on advanced imaging techniques due to the overlapping features observed in a conventional MRI [16]. Radiation necrosis can be mistaken for tumor progression which can result in unnecessary invasive procedures or suboptimal therapy [15]. Multiple advanced imaging techniques including DCE and DSC MR-perfusion, MR-spectroscopy, IVIM, and nuclear medicine studies including 18F-FET PET and 11C MET-PET have been applied to differentiate radiation necrosis from tumor progression [15]. A frequent debate has been the efficacy of radiological scans in differentiating between the two conditions. Tables 1, 2 highlight the sensitivities and specificities of each modality in identifying RN and recurrence of a cerebral tumor, respectively. 

**Table 1:** Sensitivity and specificity of different imaging modalities to assess for post-radiation necrosis MR Spectroscopy (Cho/NAA) - Magnetic Resonance Spectroscopy (Choline/N-Acetyl Aspartate), MR Spectroscopy (Cho/Cr) - Magnetic Resonance Spectroscopy (Choline/Creatine), MR Perfusion - Magnetic Resonance Perfusion, MR Spectroscopy - Magnetic Resonance Spectroscopy, PET-CT - Positron Emission Tomography - Computed Tomography, 18F-FDG-PET - Fludeoxyglucose F 18 Positron Emission Tomography, 18F-FET-PET - 18F-Fluoro-Ethyl-Tyrosine Positron Emission Tomography, 99mTc-MIBI-SPECT - 99m Technicium-Methoxyisobutylisonitrile-Single Photon Emission Computed Tomography, MRP - Magnetic Resonance Perfusion, DWI - Diffusion-Weighted Imaging, 11C-MET-PET - 11c Methionine Positron Emission Tomography, 201TI-SPECT - Thallium-201 Single-Photon Emission Computed Tomography, MRS - Magnetic Resonance Spectroscopy, Gadolinium MRI - Gadolinium Magnetic Resonance Imaging

Test	Sensitivity	Specificity	Reference
MR spectroscopy (Cho/NAA)	88%	86%	[9]
MR spectroscopy (Cho/Cr)	83%	83%	[9]
Pooled apparent diffusion coefficient	71%	83%	[9]
MR perfusion	92%	100%	[7]
MR spectroscopy	94.1%	100%	[7]
PET-CT	100%	93%	[7]
Combined imaging	96%	93%	[17]
18F-FDG-PET	81%	72%	[17]
18F-FET-PET	91%	95%	[17]
99mTc-MIBI-SPECT	92%	91%	[17]
MRP	85%	81%	[17]
DWI	88%	80%	[17]
11C-MET-PET	81%	81%	[17]
201TI-SPECT	80%	84%	[17]
MRS	83%	77%	[17]
Gadolinium MRI	63%	82%	[17]

**Table 2:** Sensitivity and specificity of different imaging modalities to assess for tumor recurrence MR Spectroscopy - Magnetic Resonance Spectroscopy, DSC-MRI - Dynamic Susceptibility Contrast Magnetic Resonance Imaging, DCE-MRI - Dynamic Contrast Enhancement Magnetic Resonance Imaging, 11C-MET-PET/CT - 11C-methyl-L-methionine PET/CT, 99m-Tc-methionine SPECT/CT - 99m-Technicium-Methionine Single Photon Emission Computed Tomography/Computed Tomography, FDG PET/CT - Fluorodeoxyglucose Positron Emission Tomography/Computed Tomography, Contrast-Enhanced MRI - Contrast-Enhanced Magnetic Resonance Imaging, SPECT - Single Photon Emission Computed Tomography

Test	Sensitivity	Specificity	Reference
MR spectroscopy	91%	95%	[18]
DSC-MRI	90%	88%	[18]
DCE-MRI	89%	85%	[18]
11C-MET-PET/CT	94.7%	88.89%	[18]
99m-Tc-methionine SPECT/CT	75.9%	90%	[18]
FDG PET/CT	82.8%	80%	[18]
Contrast-enhanced MRI	87.1%	30%	[18]
SPECT	89%	88%	[18]

From the literature search completed in this study, the highest sensitivity in ruling out RN was found to be when using a positron emission tomography-computed tomography (PET-CT), which had a sensitivity of 100%. The highest specificity seen in ruling in RN was found to be with an MRS or an MR perfusion study, both of which had a 100% specificity. In contrast, the highest sensitivity in ruling out tumor recurrence was using an 11C-MET PET/CT, which had a sensitivity of 94.7%. A study by Terakawa et al. in 2008 also examined the validity of using this tracer in differentiating between the two conditions and found that (11C-METPET/CT) was more effective in identifying tumor recurrence rather than RN [19]. A very small number of comparative studies were seen within our literature search that showed a comparison of both conditions and their respective sensitivities and specificities.

Discussion

Pertaining to the use of conventional MRI, a frequent overlap is seen between RN and tumor recurrence [18]. Both conditions typically demonstrate contrast enhancement and surrounding edema and can even co-exist. Certain enhancement patterns were thought to distinguish RN with appearances such as “Swiss Cheese”, “Soap Bubble,” or “Cut Green Pepper.” However, these signs were only noted to have a positive predictive value of 25% [7]. In the early postoperative period, roughly 10% of patients demonstrated pyriform enhancement to the brain matter, which suggests viable tumor tissue. An early thought in the differentiation of both conditions involved the use of a lesion quotient (LQ), which compared the size of a lesion on T2 sequences and the enhancing area on T1 sequences. An LQ greater than 0.6 indicated tumor recurrence and less than 0.3 indicated radiation necrosis [20]. The negative predictive values for LQ < 0.3 was 73%, and for a combination of RN and tumor recurrence (LQ between 0.3 and 0.6) was 83%, and LQ > 0.6 was 39%. This again suggested that a conventional MRI was not appropriate in differentiating between RN and the recurrence of a tumor. As evidenced in our literature review in Tables 1, 2, the sensitivity for using a conventional MRI for identifying RN and tumor recurrence was 63% and 87.1%, respectively, indicating a suboptimal standard of care for distinguishing between both conditions. 

Advanced magnetic resonance techniques, such as MR perfusion and MRS allowed for more intricate visualization of structures. MR perfusion studies, including DSC-MRI, measure the relative cerebral blood volume to distinguish blood flow to tumor tissue and necrotic tissue [7,21]. A study by Metaweh in 2018 recruited a smaller sample size of 23 patients to distinguish between both conditions and found a sensitivity of 77.8% when using DSC-MRI, which, although promising, is still suboptimal. Comparatively, a study conducted on a similar sample size by Soliman et al. showed that measuring the relative cerebral blood volume was statistically significant in distinguishing recurrent tumors and radiation necrosis and sensitivity was noted to be 87% [22]. As per our literature review, we found a similar sensitivity at 90% as seen in Table 2. This significant variability in sensitivity between the two studies highlights the importance of larger cohort studies to establish a statistically significant and generalizable set of data that would allow us to find an improved standard of care. This also brings the question as to whether multiple imaging modalities may need to be used to differentiate between both conditions as in our literature review, an MRS or MR perfusion study had 100% specificity in confirming RN whereas an 11C-METPET/CT had the highest sensitivity in ruling out recurrence of a brain tumor. 

## Conclusions

The similarities in diagnostic imaging modalities for cerebral neoplastic lesions and PRN outweigh the distinction between both entities. Among the various therapies of radiation given to patients, the clinical outcome results in almost the same identity within post-radiation apoptosis and tumor reoccurrence. However, the components of histology, cerebral volume, numerical radiation dose, and fraction size cause harm to continue due to the side effects each value measures upon the patient. Investigation to identify the slim difference in sensitivities and specificities throughout each imaging modality can aid in concluding the benefits to prevent tumor reoccurrence or continuous necrosis on neoplastic lesions. It is imperative to understand the risks and effects each imaging modality holds as it can continue to cause the cerebral tumor or further metastasize the neoplasm. Extended research must be developed to provide definite insight into thorough safety, specificity, and success in diagnostic accuracy upon neuroimaging.
